# Factors Associated with herb and dietary supplement use by young adults in the United States

**DOI:** 10.1186/1472-6882-7-39

**Published:** 2007-11-30

**Authors:** Paula Gardiner, Kathi J Kemper, Anna Legedza, Russell S Phillips

**Affiliations:** 1 Boston University Medical School, Department of Family Medicine, Boston, Massachusetts, USA; 2 Department of Pediatrics, Public Health Sciences and Family and Community Medicine, Wake Forest University School of Medicine, Winston-Salem, North Carolina, USA; 3 Division for Research and Education in Complementary and Integrative Medical Therapies, Harvard Medical School, Boston, Massachusetts, USA; 4 Department of Family Medicine, Boston University Medical Center, 1 Boston Medical Center Place, Dowling 5 South Boston, MA 02118, USA

## Abstract

**Background:**

Little is known about the association between use of herbs and dietary supplements (HDS) and lifestyle/behavior factors in young adults in the US.

**Methods:**

Analyzing the 2002 National Health Interview Survey (NHIS), we examined the patterns of HDS (excluding vitamins/minerals) use among young adults in the United States using descriptive statistics and logistic regression.

**Results:**

In our sample of 18 to 30 year olds (n = 6666), 26% were current smokers, 24% were moderate/heavy drinkers, 43% had high physical activity, and 54% and 76% use prescription and over the counter (OTC) medications respectively. Non-vitamin, non-mineral HDS was used by 17% of the overall sample in the last 12 months. In the multivariable analysis, the lifestyle and behavioral factors associated with HDS use include: current smoking (odds ratio 1.41 95% CI [1.16–1.72]); being a former smoker (1.50 [1.15–1.95]); moderate/heavy alcohol use (2.02 [1.53–2.65]); high physical activity levels (2.45 [1.98–3.03]); and prescription medication use (1.51 [1.26–1.81]). Among HDS users, only 24% discussed their use with a health care professional.

**Conclusion:**

Nearly one in five young adults report using non-vitamin/non-mineral HDS.

## Background

According to the National Institute of Health's Office of Dietary Supplements, a dietary supplement is intended to supplement the diet; and contains one or more dietary ingredients (including vitamins; minerals; herbs or other botanicals; amino acids; and other substances) or their constituents [1]. Many American adults use vitamins, minerals, botanicals, and other dietary supplements on a regular basis [1-4]. Analyses of the National Health Interview Survey (NHIS) have estimated 10 to 19 percent of the U.S. population uses a non-vitamin/mineral herb or dietary supplement (HDS) for health conditions [5-8].

Although rates of dietary supplement use vary by age, gender, race, and ethnicity, very little is known about the pattern of use among college age and young adults. Young adults are known to engage in high risk behaviors such as alcohol and tobacco use. Using certain dietary supplements, such as high dose ephedra product in a weight loss products or sport performance products is also a high risk behavior. Little is known about the correlation between high risk dietary supplements (stimulants or depressants) and other young adult's behavior.

Surveys of college athletes, college students, and young adults have demonstrated a strong link between high physical activity and use of sport supplements to enhance athletic performance [9-15]. Although this trend has been seen in special populations (i.e., athletes); it has not been replicated in a general population of young adults [9-15].

Higher rates of HDS use have also been reported among patients using prescription and non-prescription medications in pediatric and adult surveys [3]. This use is particularly concerning due to the potential for herb-medication interactions. This relationship has not been specifically evaluated in the young adult population.

Communication with health professionals about HDS use has been reported by fewer than half of adult and pediatric patients who use them [2,16,17]. This relationship has not been explored in the young adult population, but could have important implications for health care in this group.

Using data from NHIS, we examined the overall prevalence and patterns of HDS use among young adults and the prevalence of specific HDS use with lifestyle/behavioral factors such as: alcohol use, tobacco use, high physical activity, or medication use. Finally we explored disclosure rates of HDS use to health care professionals. We hypothesized that young adults would use HDS about as often as other age groups; that those engaging in high risk behaviors (alcohol and tobacco use) and athletes use HDS more often than those who do not smoke, drink alcohol or exercise regularly; that use would be higher among those using prescription and non-prescription medications. Finally, we hypothesized that fewer than half of young adults using HDS would discuss this use with a health professional.

## Methods

Previously we have described our methods of analysis [2,3]. We analyzed data from the Sample Core component and the Alternative Health Supplement to the 2002 National Health Interview Survey (NHIS). The NHIS is an in-person household survey conducted by the Census Bureau for the National Center for Health Statistics, and is the principal source of information in the U.S. on the health of the civilian, non-institutionalized household population. One adult was randomly selected from each household to complete this portion of the survey. There were 31,044 completed interviews, with a 73.4% response rate. The sampling methods for the NHIS are described elsewhere [18].

For the NHIS, respondents were specifically asked, "Some people use natural herbs for a variety of health reasons. Some people drink an herbal tea to remedy a flu or cold. Others take a daily pill to help with a health condition or just stay healthy. Have you ever used natural herbs for your own health or treatment (for example ginger, echinacea or black cohosh including teas, tinctures, and pills) [18]?" Those respondents that said yes were asked "During the past 12 months, did you use natural herbs for your own health or treatment?" For the purpose of this analysis, we will refer to any herb use as having occurred in the prior 12 months. Of those that said yes to this question, respondents were asked a series of questions about individual HDS use and disclosure of that use to conventional health care providers.

Because we were interested in high risk HDS behaviors, we excluded use of vitamins and minerals (which may have been recommended by health professionals), and focused on non-vitamin/mineral supplements. Respondents chose from a list of 35 non-vitamin/mineral supplements dietary supplements (29 supplements were plant based and 6 supplements not plant based these included: s-adenosylmethionine (SAM-e) progesterone cream, melatonin, bee pollen, fish oil, glucosamine and chondroitin).

We defined a young adult age range between 18 years of age to 30 years of age based on education and earning potential. We considered socio-demographic factors including respondents' age (18–22, 23–30) gender, education (<high school, high school graduate, some college, college graduate), annual family income (<$15,000, $15,000–34,999, $35,000–64,999, ≥ $65,000), race/ethnicity (Hispanic, non-Hispanic white, non-Hispanic black non-Hispanic other (Asian, American Indian/Alaskan native, Asian Indian, Chinese, and Filipino) and region of U.S. residence (Northeast, Midwest, South, West).

We also included lifestyle/behavioral factors that we thought could interact with or cause adverse events with HDS. These included smoking (current, former, nonsmoker); alcohol use in the past 12 months (abstainer or former drinker, current infrequent or light drinker (< 3 drinks a week), current moderate to heavy (>3 drinks per week to >14 drinks a week), or unknown drinking status);

Although physical activity level, in itself, is not a high risk behavior, adolescent and adult athletes have been reported to use herbs and other performance enhancing supplements more frequently than others of their age groups. Therefore, we analyzed a physical activity level variable [high (vigorous activity 2 times/wk or moderate activity 4 times/wk), moderate (vigorous activity 1 time/wk or moderate activity 1–3 times/wk), or sedentary (no vigorous or moderate activity/week)] in the model to assess for an association high physical activity with HDS in a national population of youth [19].

Respondents were asked if they used prescription and over the counter (OTC) medications in the last 12 months. Respondents were asked if they disclosed natural herb use to conventional medical professionals including physicians, nurse practitioners, physician assistants, psychiatrists, and dentists.

Population estimates were calculated using NHIS weights, which are calibrated to U.S. 2000 census totals for gender, age, and race/ethnicity of the 2002 U.S. population. Descriptive statistics were used to examine the prevalence of HDS use, the most common HDS used among smokers and alcohol drinkers, and disclosure to medical professionals. We used multivariate logistic regression analysis to assess which variables were significantly associated with HDS use. We selected lifestyle and behavioral variables for testing in our logistic model adjusting for age, sex, race, income, education and entered these variables into the final model simultaneously. All analyses were performed using SAS- callable SUDAAN version 8.1 (Research Triangle Institute, Research Triangle Park, NC) [20] to account for the complex sampling design of the NHIS.

## Results

Of the total 31,044 NHIS respondents, 6,666 were ages 18–30; these respondents represented approximately 49 million US young adults in 2002. The majority of young adult respondents were non-Hispanic white (64%) and age 24–30 (60%); 40% had some college experience and 19% had a college degree. (Table 1) Twenty six percent of young adults were current smokers, 32% used no alcohol, 44% were infrequent/light drinkers and 21% were moderate/heavy drinkers. Thirty four percent had low physical activity, 22% had moderate activity, and 43% had high physical activity levels. Fifty four percent used a prescription medication and 76% used an OTC medication in the last year.

**Table 1:** Demographics of Respondents Ages 18 to 30 years and the Percent of Characteristics by Individual Variables Among Those Who Use HDS

Characteristics	Total Respondents 18–30 (N = 6666) %	HDS user* (n= 1171) %	P**
***Socio-demographic variables***			
Age, yr			<.01
18–22	40	15	
23–30	60	19	
Sex			.12
Male	49	16	
Female	51	18	
Race/Ethnicity			<.01
Non-Hispanic White	64	19	
Non-Hispanic Black	14	13	
Non-Hispanic Asian	5	17	
Non-Hispanic Other***	1	27	
Hispanic	16	12	
Education Level			<.01
< High School	16	7	
High School Grad	24	15	
Some College	40	19	
College Graduate	19	25	
Income, $			<.01
<15,000	26	18	
15,000–34,999	24	21	
35,000–64,999	10	23	
≥ 65,000	2	30	
Did not reply	38	12	
***Lifestyle/behavioral variables***			
Smoking Status			<.01
Current	26	21	
Former	9	24	
Never	63	15	
Alcohol use in last 12 months			<.01
None	32	9	
Infrequent/light	44	21	
Moderate/heavy	21	24	
Unknown drinking status	3	5	
Physical Activity			<.01
Low	34	9	
Moderate	22	19	
High	43	23	
Unknown	2	6	
Prescription medication use			<.01
Yes	54	22	
No	46	13	
Over the counter medication use			<.01
Yes	76	19	
No	24	12	

* "DURING THE PAST 12 MONTHS, did you use herbs for your own health or treatment ?"

** Chi Square test comparing HDS users to non-HDS users (P < .05)

*** (Asian, American Indian/Alaskan native, Asian Indian, Chinese and Filipino)

****Data sources National health interview survey 2002, weighted percents extrapolated from the to the U.S. census (2000) for the adult civilian, non-institutionalized household population.

Of the respondents, 17% (extrapolated for the U.S. adult population to 8.4 million) reported having used a non-vitamin, non-mineral HDS in the last 12 months. Demographic factors associated with higher HDS use included (Table 1): being older, having a higher income, having a college education and being Non-Hispanic others (Asian, American Indian/Alaskan native, Asian Indian, Chinese, and Filipino). The HDS most commonly used were echinacea (used by 45% of HDS users), ginseng (34%), ginkgo biloba (22%), garlic (15%), St. Johns wort (14%), peppermint (14%), ginger (10%), chamomile, (9%), kava kava (9 %), and ephedra (7 %). The most common conditions for which HDS were used were for were head or chest cold, (reported by 40% of HDS users), stomach or intestinal conditions (12%), musculoskeletal conditions (10%), severe headache or migraine (8%), anxiety of depression (7%), and insomnia (6%).

High risk behaviors (current smoking and moderate/heavy drinking) were positively associated with HDS use. For example, rates of HDS use were 24% among former smokers, 21% among current smokers, and 15% in non-smokers. Rates of HDS use were 24% among moderate/heavy drinkers, compared with 9% in non-drinkers. Compared to non smokers, smokers used significantly more ginseng, gingko, and St. Johns wort (p < .001). Compared to non drinkers, moderate/heavy drinkers used significantly more ginseng, chamomile (p < .001 for each comparison), Echinacea (p = .04) and gingko biloba (p = .01) (Table 2).

**Table 2:** Prevalence of Specific HDS Supplement Use Among Young Adults who use Alcohol and Tobacco

	High Risk Lifestyle factors				Other Lifestyle factors	
Dietary Supplement	Smoker N = 362 %	P	Alcohol Moderate/heavy n = 335 %	P	High Physical activity N = 662 %	P

Echinacea	40	.18	49	.02	47	.002
Ginseng	42	.03	44	<.001	37	<.001
Gingko biloba	29	.02	29	.01	25	<.001
Sedating herbs valerian, melatonin, chamomile, kava kava	27	.02	28	<.001	21	.13
Garlic	17	.43	16	.54	17	.12
Peppermint	14	.79	12	.58	16	<.001
St. John's wort	20	<.001	17	.48	15	.03
Ginger	8	.09	9	.57	12	.03
Stimulant herbs ephedra, guarana	11	<.75	13	<.001	12	.005

Data sources National health interview survey 2002, weighted percents extrapolated from the to the U.S. census (2000) for the adult civilian, non-institutionalized household population.

The prevalence of HDS use was 23% for those with high physical activity, compared with for 9% low physical activity. Respondents with high physical activity used more stimulant herbs in particular (Table 2).

Use was also significantly higher among those who used prescription medications (22%) and OTC medications (18%), than among those use used neither (~13%, P < .01 for each comparison). Compared to non-prescription medication users, prescription medication users took more garlic (p = .03) and those who took OTCs used more echinacea (p = .001).

### Multivariable Analysis of Characteristics Associated with HDS Use

In the multivariable analysis, after adjusting for age, sex, race, income, and education, the factors associated with HDS use included: current smoking (odds ratio 1.41 95% CI [1.16–1.72]) former smoking (1.50 [1.15–1.95]), moderate/heavy alcohol use (2.02 [1.53–2.65]), high activity levels (2.45 [1.98–3.03]) and prescription medication use (1.51 [1.26–1.81]). OTC medication use was not associated independently with HDS use (Table 3).

**Table 3:** Independent Effects of Smoking, Alcohol use, Physical Activity, and Medication use on HDS use in Young Adults; Multivariate Logistic Regression

Lifestyle/behavioral variables	Adjusted Odds Ratios [95% confidence interval] **
Smoking Status	
Never	1.0
Current	1.41 [1.16–1.72]
Former	1.50 [1.15–1.95]
Alcohol use in last 12 months	
None	1.0
Infrequent/light	1.71 [1.38–2.13]
Moderate/heavy	2.02 [1.53–2.65]
Physical Activity	
Low	1.0
Moderate	1.88 [1.48–2.38]
High	2.45 [1.98–3.03]
Prescription medications	
No	1.0
Yes	1.51 [1.26–1.81]
Over the counter medications	
No	1.0
Yes	1.13 [.91–1.40]

*Logistic Model adjusted for age, sex, race, income, education

**Total sample size for analysis 6375, variables unknown drinking status (n= 225) and unknown physical activity (n= 97) were excluded from final analysis due to small sample size. A sensitivity analysis with the variables included showed no difference in outcomes.

***Data sources National health interview survey 2002, weighted percents extrapolated from the to the U.S. census (2000) for the adult civilian, non-institutionalized household population.

### Disclosure of HDS use to health professionals

Only 24% of those who reported using HDS disclosed their herb use to a health professional; 67% did not disclose their use, with 10% noting they did not go or talk to a conventional professional in the last 12 months. Disclosure was slightly less common among heavy drinkers than non-drinkers (18% versus 21%, P = 0.06), but similar among smokers and non-smokers and those with different activity levels. Disclosure was more common among prescription medication users than non-users (30% versus 11 %, P <0.001); it was also more common among OTC medication users compared to non- users (25% versus 17% P = 0.02)

## Discussion

Based on these data, an estimated 8 million young adults in the U.S. used a non-vitamin/mineral HDS during the prior 12 months. Use in this age group is substantial and not lower than rates in other age groups [4,5,21]. As expected, use was significantly higher among smokers, those who consume alcohol, those who reported high levels of exercise, and those who used prescription medications [22,23]. Only 24% discussed their HDS with a health care professional, a rate even lower than that seen in older adult and younger pediatric populations.

Previous studies have reported higher rates of HDS among college and university students with a prevalence ranging from of 26% to 79% [24-27]. These studies were restricted to single institutions with homogeneous populations; they also used different time periods of HDS use and used a different list of HDS [24-27]. Our rates may have been lower due to the omission of some HDS that are commonly used by athletes; for example, sport drinks, protein powders and body-building hormones were not included in the NHIS survey questions.

In our analysis, the types of individual herbs used by young adults were consistent with other studies [26,27]. Green tea, goldenseal, acidophilus, and aloe were reported as popular in previous studies but not asked about in NHIS [24].

In our multivariable analysis several lifestyle and behavioral factors were associated with HDS use. As in other studies of teenagers and adults, rates of HDS use in young adults are highest among those who are smokers and drinkers [1,5,22,23,27]. Further research is necessary to study the relationship between HDS lifestyle factors such as smoking and alcohol use and if other high risk behaviors are associated with specific herbs or supplements.

In our study, respondents with moderate and high physical activity level were more likely to use HDS. Numerous studies have found that adolescents and young adults involved in sports have a high prevalence of use dietary supplements [9,15,28-32]. The use of sport supplements is especially concerning due to their questionable safety and efficacy.

This picture becomes more complicated by reports that young adults who are athletes tend to have more alcohol and drug use [13,14]. Additionally, an association between anabolic steroid and other illicit drug use and high risk behaviors has been documented [23,33,34]. More research is necessary to understand why young athletes use HDS recreationally with alcohol or tobacco, for prevention, or for sport performance.

Among HDS users in our sample, 66% also used prescription medications and 74 % used an over the counter medication in the prior 12 months. Compared to non-prescription medication users, those who used prescription medications were more likely to use HDS. This trend has been reported in previous surveys of young adults [24,26]. Increasing numbers of reports describe clinically serious interactions between prescription drugs and herbals/supplements and concern has grown that the problem of adverse effects may be under reported [35-38].

Among young adult HDS users, the most common conditions associated with use were head or chest cold, stomach or intestinal conditions, musculoskeletal conditions, severe headache or migraine, anxiety of depression, and insomnia. In the general adult population, the most frequent conditions prompting HDS use were upper respiratory infections, depression, musculoskeletal pain, and memory improvement [4,35]. Other studies of young adults reported high use for musculoskeletal conditions, depression, weight loss, body building, allergies, gastrointestinal symptoms, and general health improvement [24-27].

In our analysis, the prevalence of talking with health professionals about HDS use (24%) is even lower than that reported in adult and young adults populations (approximately 35 to 44 %) [5,7,26,39-41]. More research is needed to understand how health care professionals and young adults discuss HDS, what benefits and adverse events young adults experience, and how best to advise them. It is critical that all health care professionals ask and counsel their patients about HDS use. For example, one study of college students, 14% reported an adverse reaction from an HDS yet 58% continued to take the supplement despite adverse reactions [26].

There are several limitations in our analysis. First, NHIS did not ask respondents about all the different types of dietary supplements used by young adults. Second, the survey is based on self-reported data, thus, subjects may be under or over-reporting their use of herbs. The term "natural herbs" (rather than herbal medications or herbal products) may have been misunderstood by respondents. Respondents who did not use natural herbs, such as sport supplement or weight lose product, may not have disclosed use. The survey listed only 29 herbs although there are thousands of botanicals sold as combination dietary supplements or ethnic traditional medicines in the U.S.

## Conclusion

In conclusion, nearly one in five young adults report using non-vitamin/non-mineral HDS; use is more common among those who smoke, drink alcohol, exercise intensively and among those who use prescription medications. More research is needed to address the safety and efficacy in the young adult population.

For patient safety, health care professionals should be aware that not only are their patients using HDS but the majority of patients are not discussing herb use with them.

## Competing interests

The author(s) declare that they have no competing interests.

## Authors' contributions

PG, KK, RP participated in the design of the study and PG, AL performed the statistical analysis. All authors read and approved the final manuscript.

## Pre-publication history

The pre-publication history for this paper can be accessed here:


